# The Evolving Challenge of Appropriate Antibiotics Use in Hospitalized COVID-19 Patients: A Systematic Literature Review

**DOI:** 10.3390/antibiotics13060545

**Published:** 2024-06-12

**Authors:** Guido Granata, Stefania Cicalini

**Affiliations:** Clinical and Research Department for Infectious Diseases, National Institute for Infectious Diseases L. Spallanzani, IRCCS, 00149 Rome, Italy

**Keywords:** COVID-19 pandemic, hospital-acquired bacterial infections, bacterial coinfection, superinfection, MDR Acinetobacter baumannii, pandrug-resistant *K. pneumoniae*, methicillin-resistant *S. aureus*, Gram-negative bacteria, antibiotic use in COVID-19 inpatients, antimicrobial stewardship, patients’ outcome, in-hospital mortality

## Abstract

The issue of bacterial infections in COVID-19 patients has received increasing attention. Scant data are available on the impact of bacterial superinfection and antibiotic administration on the outcome of hospitalized COVID-19 patients. We conducted a literature review from 1 January 2022 to 31 March 2024 to assess the current burden of bacterial infection and the evidence for antibiotic use in hospitalized COVID-19 patients. Published articles providing data on antibiotic use in COVID-19 patients were identified through computerized literature searches with the search terms [(antibiotic) AND (COVID-19)] or [(antibiotic treatment) AND (COVID-19)]. PubMed and SCOPUS databases were searched from 1 January 2022 to 31 March 2024. No attempt was made to obtain information about unpublished studies. English language restriction was applied. The quality of the included studies was evaluated by the tool recommended by the Joanna Briggs Institute. Both quantitative and qualitative information were summarized by means of textual descriptions. Five hundred fifty-one studies were identified, and twenty-nine studies were included in this systematic review. Of the 29 included studies, 18 studies were on the prevalence of bacterial infection and antibiotic use in hospitalized COVID-19 patients; 4 studies reported on the efficacy of early antibiotic use in COVID-19; 4 studies were on the use of sepsis biomarkers to improve antibiotic use; 3 studies were on the efficacy of antimicrobial stewardship programs and predictive models among COVID-19-hospitalized patients. The quality of included studies was high in 35% and medium in 62%. High rates of hospital-acquired infections were reported among COVID-19 patients, ranging between 7.5 and 37.7%. A high antibiotic resistance rate was reported among COVID-19 patients developing hospital-acquired infections, with a high in-hospital mortality rate. The studies evaluating multi-faceted antimicrobial stewardship interventions reported efficacy in decreasing antibiotic consumption and lower in-hospital mortality.

## 1. Introduction

The use of antibiotics among COVID-19 patients and the risk of bacterial infections has become a growing concern [1]. Hospitalized COVID-19 patients are at risk of developing bacterial superinfections during their stay [1]. Currently, there are limited data available on the impact of bacterial superinfections and antibiotic administration on hospitalized COVID-19 patients [1,2].

Reports suggest that bacterial co-infections in COVID-19 patients can occur at rates ranging from 1.2% to 43% [1,2]. However, the effectiveness of administering antimicrobials to hospitalized COVID-19 patients in reducing length of stay and all-cause mortality is not yet clear [1]. Clinically, it is often challenging to differentiate between bacterial and viral pneumonia [3]. Moreover, the practice of administering broad-spectrum antibiotics to those with COVID-19 could inadvertently lead to the emergence of bacteria resistant to multiple drugs [4].

We conducted a literature review from 1 January 2022 to 31 March 2024 to assess the current burden of bacterial infection and the evidence of antibiotic use in hospitalized COVID-19 patients.

## 2. Materials and Methods

The study was conducted according to the Preferred Reporting Items for Systematic Reviews and Meta-Analyses (PRISMA) guidelines, 2020 version (Supplementary Materials, Tables S1 and S2). The study protocol has been registered on the International Prospective Register of Systematic Reviews (PROSPERO); registration code: CRD42024547272.

### 2.1. Search Strategy and Article Identification

Scientific articles on bacterial infections in COVID-19 patients and the use of antibiotics to treat COVID-19 patients were searched on the MEDLINE and SCOPUS databases from 1 January 2022 to 31 March 2024.

Searching the MEDLINE database, the following search terms were used: (antibiotic) AND (COVID-19). Filters applied: Clinical Study, from 2022 to 2024. 

Searching the SCOPUS database, the following search terms were used: [(antibiotic treatment) AND (COVID-19)]. The following filters were applied: Subject area: Medicine; Document type: Article; Keyword: Hospital patients; Publication date: from 2022 to 2024.

Supplementary Figure S1 provides details on the query specifications used for the MEDLINE and SCOPUS databases (Supplementary Figure S1).

No attempt was made to gather data from unpublished research. Studies that were only published as abstracts, correction articles, review articles, case reports, editorials, and clinical trial protocols were not considered for further evaluation.

### 2.2. Eligibility Criteria

Original articles, meta-analyses, and randomized clinical trials reporting data on bacterial infections in adult COVID-19 patients and the use of antibiotics to treat adult COVID-19 patients were eligible for inclusion.

### 2.3. Study Selection, Data Extraction, and Quality Appraisal

Eligibility assessment and extraction of data were performed independently by two investigators. Each investigator had no access to the other investigator’s data extraction. The quality of the included studies was evaluated by the tool recommended by the Joanna Briggs Institute [5], considering 9 items (with a dichotomic response, yes/no), as shown in Table S3. In case of fulfilment of a specific criterium, 1 point was attributed, thereby entailing a total score ranging from 0 to 9. Studies were deemed of high, medium, and low quality if the total score was >7 points, 6–7 points, and <6 points, respectively [6]. In case of disagreement between the two reviewers, a third external expert in the field was consulted.

### 2.4. Data Synthesis

For the data syntheses, included articles were grouped into four groups: studies on the prevalence of bacterial infection and antibiotic use in hospitalized COVID-19 patients; studies on the efficacy of early antibiotic use in hospitalized COVID-19 patients; studies on the use of sepsis biomarkers to improve antibiotic use and studies on the efficacy of antimicrobial stewardship programs among COVID-19 inpatients. Both quantitative and qualitative information were summarized using textual descriptions.

## 3. Results

### 3.1. Study Descriptions

**Figure 1:**
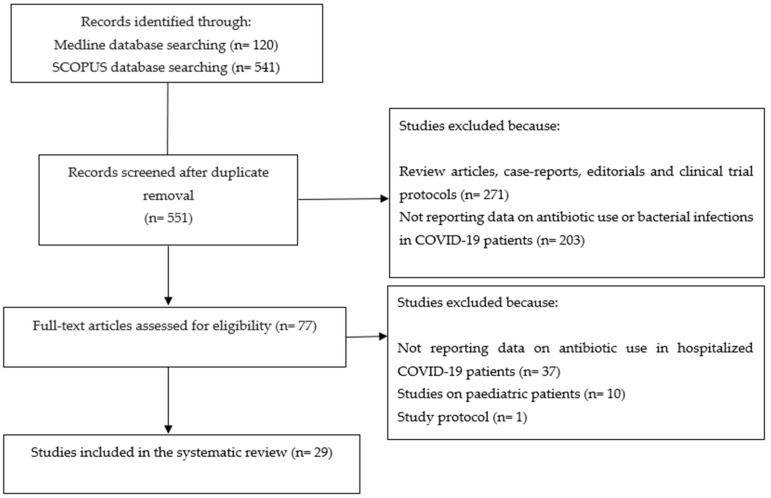
Flowchart depicting the selection process of studies included in the systematic review.

Figure 1 shows the selection process of studies included in the review. 

Through the database searches, we identified 551 studies. Two hundred seventy-one studies were excluded because of review articles, case reports, editorials, and clinical trial protocols. Two hundred-three studies were excluded because they did not report data on antibiotic use in COVID-19 patients. Of the remaining 77 studies, 29 studies were included in this systematic review [7,8,9,10,11,12,13,14,15,16,17,18,19,20,21,22,23,24,25,26,27,28,29,30,31,32,33,34,35] (Table 1). 

Of the 29 studies included in the systematic review, 18 studies were on the prevalence of bacterial infection and antibiotics use in hospitalized COVID-19 patients [7,8,9,10,11,12,13,14,15,16,17,18,19,20,21,22,23,24]; of these studies, 5 reported on patients admitted to the intensive care unit [20,21,22,23,24]. Four studies reported on the efficacy of early antibiotic use in hospitalized COVID-19 patients [25,26,27,28]; four studies were on the use of sepsis biomarkers to improve antibiotic use among COVID-19 patients [29,30,31,32]; three studies were on the efficacy of antimicrobial stewardship programs and predictive models among COVID-19 hospitalized patients [33,34,35].

Among the 29 included studies, 23 studies were retrospective, observational studies [7,8,9,10,11,12,13,14,15,16,17,18,19,20,21,22,23,24,26,27,29,30,35]; 4 studies were observational, before–after studies [28,32,33,34]; and 2 studies were randomized, open-label, controlled trials [25,31].

A summary description of the included studies is reported in Supplementary Tables S4 and S5, as well as Tables S6 and S7.

The quality of included studies was high in 35% and medium in 62% of the studies. Major factors limiting quality were inadequate participant recruitment, inadequate distinction between colonization and actual bacterial infection, and an inadequate sample size.

### 3.2. Bacterial Infections and Antibiotic Use among COVID-19 Patients

#### 3.2.1. The Prevalence of Bacterial Infection and Antibiotic Use among Hospitalized COVID-19 Patients

An observational cohort study was conducted to evaluate the prevalence of bacterial infection and antibiotic administration among 717 hospitalized COVID-19 patients. Of the total patients, 12.0% (86/717) received antibiotics but only 3.6% (26/717) had a documented bacterial infection. The majority of patients were treated with amoxicillin–clavulanate (76.0%), piperacillin–tazobactam (28.0%), macrolides (46.7%), carbapenems (18.7%), cephalosporins (14.7%), and fluoroquinolones (8.0%). Among the 278 patients with COVID-19 pneumonia, those who received antibiotic treatment exhibited a higher incidence of diarrhoea (34.7% versus 11.8%, *p* < 0.01). However, subsequent admissions to the intensive care unit were not lower (8.0% versus 4.9%, *p*: 0.384). Antibiotic treatment was not independently associated with lower 30-day or in-hospital mortality rates [7].

A retrospective study was conducted to identify the risk factors associated with antibiotic use and to evaluate the impact of antimicrobial therapy on COVID-19 patients. Of the 164 patients, 100 (61%) received antibiotic treatment, while 28 (17.1%) had a confirmed infection, mostly in the urinary tract (18/28, 64.3%). The isolates were mostly Gram-negative bacteria, including *E. coli* (15/29, 51.7%), *P. aeruginosa* (3/29, 10.3%), *K. pneumoniae* (3/29, 10.3%), and *C. koseri* (2/29, 6.9%). Six factors were associated with antibiotic use: being hospitalized in the intensive care unit [odds ratio (OR): 4.59; 95% confidence interval (CI): 1.07–19.71), age > 65 years (OR: 4.16; 95% CI: 1.72–10.05), arrival from a nursing home (OR: 4.59; 95% CI: 1.11–19.71), diabetes (OR: 4.35; 95% CI: 1.26–14.93), radiological chest bilateral consolidation (OR: 9.92; 95% CI: 2.40–41.06), and a C-reactive protein level over 60 mg/L (OR: 2.46; 95% CI: 1.13–5.37). Antibiotic treatment did not reduce the length of stay or the mortality rate [8].

A cohort study evaluated the prevalence of bacterial bloodstream infection in COVID-19 patients during the first wave of the pandemic. The study included 595 COVID-19 patients. The prevalence of bloodstream infection was 4.2% (25/595 patients) over a 28-day period. Patients with bloodstream infection had a higher 30-day mortality rate (68% versus 34%, *p* < 0.01) [9].

A retrospective study was performed during the first wave of the pandemic to describe the incidence, aetiology, and outcome of blood or respiratory tract bacterial infections. The study involved 585 hospitalized COVID-19 patients; 40/585 (6.8%) developed lung or bloodstream bacterial infection. These patients had longer hospital stays in comparison to patients without bacterial infection (31 versus 9 days; *p* < 0.001) [10].

A retrospective study was conducted on 7249 COVID-19 patients who were hospitalized in Serbia. The study aimed to describe the aetiology of bacterial infections, antibiotic resistance patterns, treatment approaches, risk factors, and mortality rates. The prevalence of bacterial infections was 12.9%, with hospital-acquired infections being the most common (11.5%). Notably, Gram-negative bacteria (*n*: 977, 69.3%) were more prevalent than Gram-positive bacteria (*n*: 433, 30.7%). The most common hospital-acquired infections were bloodstream infections (37.7%) and pneumonia (25.6%). Urinary tract infections, gastroenteritis, and skin and soft tissue infections were also reported. *K. pneumoniae* and *A. baumannii* caused 25.2% and 23.6% of all bacterial infections, respectively. The reported prevalence of multidrug-resistant (MDR), extensively drug-resistant, and pandrug-resistant bacteria were 24.2% (*n*: 341), 37.9% (*n*: 534), and 12.8% (*n*: 180), respectively. The overall in-hospital mortality rate of COVID-19 patients with bacterial infections was 51.6% [11].

A retrospective study evaluated antibiotic use rates in hospitalized COVID-19 patients. Out of 198 patients, 83% received at least one course of antibiotics despite relatively low rates of microbiologically confirmed infection (12%) [12]. 

A large cohort study in the US compared antibiotic prescribing trends for COVID-19 patients, categorized by disease severity, with a non-COVID-19 population experiencing similar symptoms. The COVID-19 patient population included approximately 1.8 million patients. In the COVID-19 cohorts, approximately 11% of patients received an antibiotic prescription. Among patients with antibiotic prescriptions, about 37% were prescribed an antibiotic “appropriately”, 39% were prescribed a “potentially appropriate” antibiotic, and about 23% received an “inappropriate” antibiotic [13].

The retrospective study by Di Lorenzo et al. described antibiotic prescription practice in COVID-19 patients before and after hospitalization. Bacterial infections at admission among COVID-19 hospitalized patients were rare and not associated with higher mortality [14]. 

A retrospective study at two hospitals in Pakistan assessed the prevalence of methicillin-resistant *S. aureus* (MRSA) among COVID-19 patients. Among the 3492 included patients, the prevalence of MRSA was 7.33% in patients aged ≥ 50 years [15].

A retrospective, propensity-matched cohort study was conducted using the US National COVID Cohort Collaborative database to investigate temporal trends and outcomes associated with prescribing antibiotics early to hospitalized COVID-19 patients. Out of the 322,867 hospitalizations, 43,089 patients (13.3%) received early antibiotics. The authors concluded that the frequency of empiric intravenous antibiotic treatment for COVID-19 patients declined during the pandemic. However, its use remains higher than the reported incidence of bacterial superinfection, with significant inter-centre variation in antibiotic prescribing practices [16].

A retrospective cross-sectional study described antibiotic resistance rates among 2786 adult COVID-19 patients, adopting a laboratory-based surveillance approach. The prevalence of bacterial infection among COVID-19 patients was 16.4%, predominating Gram-negative bacteria. *K. pneumoniae, A. baumannii*, and *P. aeruginosa* were commonly identified in the respiratory specimens. High resistance to ampicillin–sulbactam (24–100%), ceftriaxone (22–81%), cefotaxime (22–73%), and ciprofloxacin (20–86%) were reported [17].

Furthermore, a cohort study observed the incidence and consequences of potential secondary infections in elderly COVID-19 patients who were hospitalized. The study comprised 266 COVID-19 patients with an average age of 81 years, of whom 115 (43%) developed a bacterial infection. Patients who contracted bacterial infections were more likely to die (45.2% versus 30.7%, *p*: 0.02) and had a longer hospital stay (23 versus 18 days, *p*: 0.026) [18].

Finally, a retrospective cohort study aimed to characterize bacterial infections and antibiotic use among hospitalized COVID-19 patients with cancer. Of 358 patients, 234 (65%) had a solid cancer. The proportion of patients with bacterial infection increased with COVID-19 severity: mild 35%, moderate 51%, severe 81% (*p*: 0.0001). The five most frequently administered antibiotics were piperacillin–tazobactam, cefepime, ceftriaxone, azithromycin, and meropenem [19].

#### 3.2.2. The Prevalence of Bacterial Infection and Antibiotic Use among Hospitalized COVID-19 Patients in the Intensive Care Unit

An observational, retrospective study described the incidence, outcomes, and risk factors of ventilator-associated pneumonia recurrences among COVID-19 patients requiring mechanical ventilation. Of the 398 patients included, 236 (59%) had ventilator-associated pneumonia, and 109/236 (46%) of them developed at least one pneumonia recurrence. Patients who experienced a recurrence of ventilator-associated pneumonia had a longer stay in the intensive care unit (46 days compared to 22 days; *p* < 0.001) and a higher 90-day mortality rate compared to those who did not develop ventilator-associated pneumonia (31% versus 21%; *p*: 0.021) [20].

Similarly, a retrospective study assessed the prevalence of bacterial pulmonary infections among patients with severe COVID-19 pneumonia in a Moroccan intensive care unit. A large proportion of patients (68%) received antibiotics before intensive care unit admission. Overall, intensive care unit mortality was 64.5%, and patients with bacterial pneumonia showed a higher risk of death (OR: 6.4, 95% CI: 1.8–22; *p*: 0.004). The prevalence of healthcare-associated pneumonia and ventilator-associated pneumonia was 12% and 40%, respectively (64 ventilator-associated pneumonia/1000 ventilation days). The most commonly isolated pathogens were *Enterobacterales* and *Acinetobacter* spp., with a proportion of MDR of 78% for *Acinetobacter* spp. and 24% for *Enterobacterales* [21].

Similarly, an observational cohort study on 29 critically ill patients requiring mechanical ventilation due to COVID-19 reported a higher mortality rate in patients with bacterial infection (81% versus 25%, *p*: 0.009) [22].

A single-centre, retrospective study on 73 COVID-19 patients admitted to the intensive care unit and developing carbapenem-resistant *A. baumannii* infections reported 80.8% mortality at 30 days [23].

A study reported an increasing trend in the incidence of infections due to MDR Gram-negative bacteria in an intensive care unit during the first wave of the pandemic compared to the pre-pandemic period [24].

#### 3.2.3. The Efficacy of Early Antibiotic Administration in COVID-19

A randomized, non-blinded trial enrolled 387 COVID-19 patients to test the hypothesis that doxycycline is effective in preventing intensive care unit admission in hospitalized COVID-19 patients. Doxycycline was reported as associated with a relative risk reduction (31.6%) for intensive care unit admission [25].

A multicentre, retrospective cohort study evaluated the effect of early antibiotic administration on COVID-19-hospitalized patients without bacterial infection. The study analysed 1373 patients who were divided into two groups based on their exposure to antibiotics within 48 h of admission. During the 30-day follow-up period, a higher proportion of patients in the early antibiotic use group progressed to severe COVID-19 compared to the comparison group. In the subgroup analysis, it was found that azithromycin did not improve disease progression or length of stay [26].

A retrospective cohort study analysed 4462 COVID-19 patients to assess the advantages and disadvantages of administering azithromycin within 24 h of hospital admission, compared to standard care. The study found that azithromycin treatment was consistently linked to a higher risk of acute heart failure in patients with pre-existing cardiovascular disease (risk ratio: 1.48, 95% CI: 1.06–2.06) [27].

Additionally, a pre-post observational study was conducted to evaluate the effectiveness of selective digestive decontamination in reducing the incidence of ventilator-associated pneumonia in COVID-19 patients. The decontamination involved the application of a suspension containing tobramycin sulfate, colistin sulfate, and amphotericin B to the patient’s oropharynx and stomach via a nasogastric tube. The study included 348 adult COVID-19 patients who required invasive mechanical ventilation. The study found that the incidence of ventilator-associated pneumonia decreased by 7.7% among the 86 patients who received selective digestive decontamination [28].

#### 3.2.4. Studies on the Use of Sepsis Biomarkers for Antibiotic Prescribing in COVID-19-Hospitalized Patients

The retrospective study by Conlon et al. evaluated 793 COVID-19-hospitalized patients to describe the natural course of procalcitonin in COVID-19 patients. Multivariable models were used to assess associations between procalcitonin level and bacterial pneumonia with antimicrobial use. Of the 793 patients, 224 (28.2%) were initiated on antibiotics: 33 (14.7%) had proven or probable bacterial pneumonia, 125 (55.8%) had possible bacterial pneumonia, and 66 (29.5%) had no bacterial pneumonia. Among patients receiving antibiotics, the initial median procalcitonin level was 0.20 ng/mL for those with no bacterial pneumonia, 0.65 ng/mL for those with possible bacterial pneumonia, and 0.88 ng/mL for those with probable or proven bacterial pneumonia [29].

A cohort study evaluated the use of a procalcitonin-guided antibiotic protocol to reduce antibiotic use in COVID-19 patients. The study compared 1335 COVID-19 patients in terms of antibiotic consumption. The decision to treat patients with antibiotics was guided by procalcitonin levels. Antibiotics were discouraged for procalcitonin levels below 0.25 ng/mL, could be considered for levels between 0.25 and 0.5, and recommended for levels above 0.5. During the same period, one group of 216 patients was treated based on a procalcitonin algorithm, while two control groups of 57 and 486 patients did not undergo procalcitonin measurements. During the first 7 days, antibiotic prescription was 26.8% in the procalcitonin group, 43.9% in the non-procalcitonin group in the same hospital, and 44.7% in the non-procalcitonin group in other hospitals. The odds of receiving antibiotics in the first 7 days of admission were lower for patients in the procalcitonin group (OR: 0.33; 95% CI: 0.16–0.66). During the entire admission, 35.2%, 43.9%, and 54.5% of patients received antibiotic prescriptions, respectively. There were no significant differences in other secondary endpoints [30].

A multicentre, parallel-group, open-label, randomized controlled trial performed among 13 intensive care units in France included 194 critically ill COVID-19 patients to assess the efficacy and safety of antibiotic exposure due to a strategy combining a respiratory multiplex polymerase chain reaction panel and daily procalcitonin measurements. Patients were assigned (1:1) to the control strategy, in which antibiotic streamlining remained at the discretion of the physicians, or interventional strategy, consisting of using multiplex polymerase chain reaction panel and daily procalcitonin measurements within the first 7 days of randomization to streamline initial antibiotic therapy, with antibiotic continuation encouraged when procalcitonin was >1 ng/mL and discouraged if <1 ng/mL or decreased by 80% from baseline. The trial found that respiratory bacterial coinfection was detected in 48.4% (45/93) and 21.4% (21/98) in the interventional and control group, respectively. No significant difference was observed in the number of antibiotic-free days (12 versus 14 days, respectively; *p:* 0.89). Mortality rates and intensive care unit lengths of stay did not differ between groups [31]. 

Furthermore, a multicentre quasi-experimental study assessed the effectiveness of using procalcitonin levels and the ‘clinical pulmonary infection’ score in reducing inappropriate antibiotic use and the incidence of multidrug-resistant bacteria among COVID-19 patients. The study involved 406 COVID-19 patients who were hospitalized. The study was conducted over a period of two years, with a one-year pre-implementation period and a one-year post-implementation period. During the implementation period, upon admission, the researchers calculated the ‘clinical pulmonary infection’ score and ordered admission procalcitonin for all patients. For those with a score of less than 6 and a procalcitonin level of less than 0.5 μg/L, no antibiotics were initiated. At 3 days, the procalcitonin and clinical pulmonary infection scores were reassessed. If the patient’s score was less than 6 and the procalcitonin level was less than 0.5 μg/L or less than 80%, antibiotics were discontinued. The study found a significant reduction in inappropriate antibiotic use, from 63.5% to 31.3% (*p* < 0.01), following the implementation of the protocol. Additionally, the incidence of MDR bacteria, including *A. baumannii*, was significantly lower (*p*: 0.04), and there was a shorter total antibiotic duration (7 versus 0 days, *p* < 0.01) and length of stay (13 versus 10 days, *p* < 0.01). The 30-day mortality was not significantly different [32].

#### 3.2.5. Studies on the Efficacy of Antimicrobial Stewardship Programs

A recent before–after study assessed the effects of a multiphase antimicrobial stewardship intervention in COVID-19 wards during the pandemic. The study compared antimicrobial usage during the three waves of the pandemic to the 12-month period before the pandemic. Antimicrobial stewardship measures included having an infectious disease specialist present during unit clinical rounds, a biweekly review of ongoing antibiotic therapies, dissemination of COVID-19 guidelines through a mobile app, and prospective weekly audits. These interventions led to a decrease in total antibiotic consumption over time and had a positive impact on the ‘watch’ antibiotics class and the use of piperacillin–tazobactam (*p* < 0.05) [33].

During the COVID-19 pandemic, a retrospective–prospective, quasi-experimental, before–after study was conducted to evaluate carbapenem-focused antimicrobial stewardship. A multi-faceted antimicrobial stewardship intervention was implemented, whereby the pharmacy alerted the infectious diseases specialist upon prescription order for a carbapenem, who then provided unsolicited consultation within 72 h. The study found that the proportion of admitted patients who received carbapenems decreased significantly from 4.1% to 2.3% (*p* < 0.001). During the post-implementation period, the acceptance of the antimicrobial stewardship intervention was linked to a lower daily hazard of in-hospital death (hazard ratio: 0.49, 95% CI: 0.30–0.80), lower odds of 30-day mortality (OR: 0.36, 95% CI: 0.18–0.70), and a higher rate of treatment success (hazard ratio: 2.45, 95% CI: 1.59–3.77) [34].

Recently, Giannella M et al. built a predictive model to stratify the risk of bacterial coinfection in hospitalized COVID-19 patients. The study included 1733 COVID-19 patients. At multivariable analysis in the derivation cohort, white blood cells  ≥  7.7/mm^3^, procalciton  ≥ 0.2 ng/mL, and Charlson index  ≥ 5, were risk factors for bacterial coinfection. In the validation cohort, the predictive model showed a positive predictive value of 16.0% and a negative predictive value of 97.5% [35].

## 4. Discussion

The aim of our systematic review was to evaluate the available evidence on the use of antibiotics in COVID-19 inpatients. 

Our data showed that antibiotics were widely used, ranging from 12% to 83%, despite only a small percentage of COVID-19 patients receiving antibiotics having a documented bacterial infection (3.6%–17.0%) [7,8,12,16]. Analysing the data from the included studies, a significant variation in antibiotic prescribing practices emerges between different hospitals and centres.

In previous studies conducted before 2022, bacterial coinfections were reported in limited proportions among COVID-19 patients upon admission, and few patients developed superinfections during hospitalization [1]. Conversely, upon evaluation of the studies conducted over the past two years, a high prevalence of hospital-acquired infections was found, specifically bloodstream infections ranging from 7.5% to 37.7% and bacterial pneumonia ranging from 4.2% to 25.6% [9,10,11]. Additionally, a concerning rate of antibiotic resistance has been observed among COVID-19 patients who develop hospital-acquired infections, with carbapenem resistance reaching up to 69.1% Gram-negative isolates [11]. 

Regarding the intensive care unit setting, the picture is alarming, with high rates of bloodstream infections and ventilator-associated pneumonia due to MDR bacteria and high mortality rates [11,21,22]. These findings highlight the urgent need to increase antimicrobial stewardship in intensive care units [21]. 

One possible explanation for the reported higher rate of hospital-acquired infections among COVID-19-hospitalized patients over the past two years is that during the initial wave of the pandemic, younger patients with fewer underlying health conditions were admitted to hospitals. Currently, patients hospitalized for COVID-19 are often frail and have multiple underlying health conditions, resulting in severe cases of COVID-19. These patients frequently require extended hospital stays and admission to the intensive care unit, which increases the risk of developing hospital-acquired bacterial infections. 

Moreover, the highest rates of antibiotic resistance observed in the setting of intensive care units may be explained as it is more challenging to observe infection control procedures and antimicrobial stewardship in this setting.

Of note, a high prevalence of community-acquired bacterial infection has been noted in studies evaluating specific patients’ groups, i.e., a 66% prevalence of community-acquired pneumonia among cancer patients hospitalized with COVID-19 [19] and a 43% prevalence of bacterial infection within 14 days from COVID-19 diagnosis in a cohort of elderly patients [18]. We believe that these findings do not alter the advice to avoid antibiotics in most cases of mild COVID-19. However, they emphasize the need for a comprehensive assessment of other potential infection sources in specific high-risk patient groups, such as elderly patients, patients with cancer, or immunodeficiency [18].

A wider dissemination of guidelines on COVID-19 management could be beneficial. Guidelines recommend collecting cultures before administering antibiotics and assessing therapy daily for de-escalation or discontinuation [36,37]. Antibiotics should be discontinued when microbiology cultures are negative [36,37]. 

Under these considerations, there is significant interest in the data from studies on sepsis biomarkers for antibiotic prescribing, as well as the effectiveness of antimicrobial stewardship programs and predictive models in COVID-19-hospitalized patients.

However, the four studies included in this analysis that evaluated the use of procalcitonin to guide antibiotic administration produced conflicting results [29,30,31,32]. Conlon et al. observed that, despite the low prevalence of bacterial coinfection at presentation, patients with COVID-19 may have elevated procalcitonin levels [29]. Therefore, considering that COVID-19 can raise the procalcitonin level in the absence of bacterial coinfection, the procalcitonin threshold may need to be increased to >0.25 ng/mL when assessing the probability of bacterial coinfection with COVID-19 [29]. The encouraging data reported by a study adopting a combined approach using repeated procalcitonin assessment and clinical score calculation sounds promising for the reduction of instances of inappropriate antibiotic administration [32].

Importantly, the available studies evaluating the efficacy of multi-faceted antimicrobial stewardship interventions in COVID-19 wards reported favourable outcomes, with a decreased amount of total antibiotic consumption, lower in-hospital mortality, and a higher rate of treatment success [33,34]. Interestingly, Giannella M et al. reported the development of a predictive model to stratify the risk of bacterial infection in hospitalized COVID-19 patients. The model showed a negative predictive value of 97.5%, which may help to reduce inappropriate antibiotic use in the future [35].

One limitation of our review is that the included studies were highly diverse, conducted in various settings, and had different objectives. It is possible that the new knowledge gained since the start of the COVID-19 pandemic has led to changes in antibiotic use. Furthermore, there was variation in the definition of bacterial coinfection and superinfection and of multi-drug resistance, with some studies providing insufficient detail. Acknowledging these pitfalls, our findings confirm the crucial role of antimicrobial stewardship and infection control in wards and intensive care units admitting COVID-19 patients.

In conclusion, our systematic review confirms that the frequency of antibiotic prescriptions remains higher than the reported incidence of bacterial coinfection at admission. The incidence of hospital-acquired bacterial superinfections during the hospitalization of COVID-19 patients is on the rise, along with the prevalence of MDR infections that have a high mortality rate. Further evidence is required to determine the effectiveness of procalcitonin-guided antibiotic prescription in reducing antibiotic prescription rates without significant safety concerns. The implementation of multi-faceted antimicrobial stewardship programs and infection control can significantly improve the antibiotic use and outcome of COVID-19 patients.

## Figures and Tables

**Table 1:** Description of the main findings of the included studies on bacterial infections in COVID-19 patients and the use of antibiotics to treat COVID-19 patients.

Main Issue Evaluated by the Included Studies	Number of Included Studies	Main Findings	Comments
The prevalence of bacterial infection and antibiotic use in hospitalized COVID-19 patients	18	Antibiotic administration ranged between 12% and 83%, despite only a small percentage of the patients receiving antibiotics had a documented bacterial infection. Rates of hospital-acquired infections range between 7.5% and 37.7%. Hospital-acquired bacterial pneumonia ranges between 4.2% and 25.6% High rates of MDR Gram-negative bacteria and high in-hospital mortality rate of COVID-19 patients with infection due to MDR bacteria, up to 51.6%	High antibiotic resistance rate has been described among COVID-19 patients developing hospital-acquired infections, with carbapenem-resistance in more than 69.1% of the Gram-negative isolates. In intensive care units, high levels of bacterial superinfections, in particular, MDR Gram-negative bacteria burdened with high mortality rates. Higher bacterial infection rate in specific patients’ groups, i.e., 67% of cancer patients and 43% of elderly patients with COVID-19
The efficacy of early antibiotic use	4	Azithromycin does not improve disease progression and length of stay in COVID-19 patients	Early antibiotic use does not improve disease progression and length of stay
The use of sepsis biomarkers to improve antibiotic use	4	The included studies evaluating procalcitonin-guided antibiotic prescription to reduce antibiotic prescription rates in hospitalized COVID-19 patients gave conflicting results	Considering that COVID-19 can raise the procalcitonin level in the absence of bacterial coinfection, procalcitonin threshold may need to be increased to >0.25 ng/mL when assessing the probability of bacterial coinfection with COVID-19. A combined approach using procalcitonin and clinical score sounds promising
The efficacy of antimicrobial stewardship programs	3	Decreased amount of total antibiotic consumption and lower in-hospital mortality after the implementation of multi-faceted antimicrobial stewardship interventions in COVID-19 wards	Predictive models to stratify the risk of bacterial coinfection in hospitalized COVID-19 patients may be implemented into antimicrobial stewardship interventions

## Data Availability

The original data presented in the study are openly available on the MEDLINE and SCOPUS databases.

## Supplementary Materials

The following supporting information can be downloaded at: https://www.mdpi.com/article/10.3390/antibiotics13060545/s1, Supplementary Figure S1: Search strategy through electronic databases. Table S1: PRISMA 2020 for Abstracts Checklist. Table S2: PRISMA 2020 Checklist. Table S3. Quality appraisal of the included studies. Table S4. Summary description of the studies on the prevalence of bacterial infection and antibiotics use in hospitalized COVID-19 patients included in the systematic review. Table S5. Summary description of the studies on the efficacy of early antibiotic administration in COVID-19 patients. Table S6. Summary description of the studies on the use of sepsis biomarkers to improve antibiotic use in COVID-19 hospitalized patients. Table S7. Summary description of the studies on the efficacy of antimicrobial stewardship programs in COVID-19-hospitalized patients.
