# Supplementary material for: The Evolving Challenge of Appropriate Antibiotics Use in Hospitalized COVID-19 Patients: A Systematic Literature Review
Source: Antibiotics (Basel). 2024 Jun 12;13(6):545. doi: 10.3390/antibiotics13060545 (PMC11200443; doi:10.3390/antibiotics13060545)
Supplement: Supplementary file 1 [file antibiotics-13-00545-s001.zip › antibiotics-3034841-supplementary.pdf]

antibiotics

MDPI

Supplementary Materials

The Evolving Challenge of Appropriate Antibiotics Use in Hospitalized COVID-19 Patients: A Systematic Literature Review

Guido Granata and Stefania Cicalini

Table S1. PRISMA 2020 for Abstracts Checklist.

Section and Topic	Item #	Checklist item	Reported (Yes/No)
**TITLE**			
Title	1	Identify the report as a systematic review.	Yes
**BACKGROUND**			
Objectives	2	Provide an explicit statement of the main objective(s) or question(s) the review addresses.	Yes
**METHODS**			
Eligibility criteria	3	Specify the inclusion and exclusion criteria for the review.	Yes
Information sources	4	Specify the information sources (e.g. databases, registers) used to identify studies and the date when each was last searched.	Yes
Risk of bias	5	Specify the methods used to assess risk of bias in the included studies.	Yes
Synthesis of results	6	Specify the methods used to present and synthesise results.	Yes
**RESULTS**			
Included studies	7	Give the total number of included studies and participants and summarise relevant characteristics of studies.	Yes
Synthesis of results	8	Present results for main outcomes, preferably indicating the number of included studies and participants for each. If meta-analysis was done, report the summary estimate and confidence/credible interval. If comparing groups, indicate the direction of the effect (i.e. which group is favoured).	Yes
**DISCUSSION**			
Limitations of evidence	9	Provide a brief summary of the limitations of the evidence included in the review (e.g. study risk of bias, inconsistency and imprecision).	Yes
Interpretation	10	Provide a general interpretation of the results and important implications.	Yes
**OTHER**			

Section and Topic	Item #	Checklist item	Reported (Yes/No)
Funding	11	Specify the primary source of funding for the review.	n/a
Registration	12	Provide the register name and registration number.	n/a

For more information, visit: http://www.prisma-statement.org/.

Table S2. PRISMA 2020 Checklist.

Section and Topic	Item #	Checklist item	Location where item is reported
**TITLE**			
Title	1	Identify the report as a systematic review.	Line 1-3
**ABSTRACT**			
Abstract	2	See the PRISMA 2020 for Abstracts checklist.	Line 10-32
**INTRODUCTION**			
Rationale	3	Describe the rationale for the review in the context of existing knowledge.	Line 39-50
Objectives	4	Provide an explicit statement of the objective(s) or question(s) the review addresses.	Line 51-52
**METHODS**			
Eligibility criteria	5	Specify the inclusion and exclusion criteria for the review and how studies were grouped for the syntheses.	Line 72-90
Information sources	6	Specify all databases, registers, websites, organisations, reference lists and other sources searched or consulted to identify studies. Specify the date when each source was last searched or consulted.	Line 59-63
Search strategy	7	Present the full search strategies for all databases, registers and websites, including any filters and limits used.	Line 62-66
Selection process	8	Specify the methods used to decide whether a study met the inclusion criteria of the review, including how many reviewers screened each record and each report retrieved, whether they worked independently, and if applicable, details of automation tools used in the process.	Line 76-84
Data collection process	9	Specify the methods used to collect data from reports, including how many reviewers collected data from each report, whether they worked independently, any processes for obtaining or confirming data from study investigators, and if applicable, details of automation tools used in the process.	Line 76-91
Data items	10a	List and define all outcomes for which data were sought. Specify whether all results that were compatible with each outcome domain in each study were sought (e.g. for all measures, time points, analyses), and if not, the methods used to decide which results to collect.	Line 86-91
	10b	List and define all other variables for which data were sought (e.g. participant and intervention characteristics, funding sources). Describe any assumptions made about any missing or unclear information.	Line 86-91
Study risk of bias assessment	11	Specify the methods used to assess risk of bias in the included studies, including details of the tool(s) used, how many reviewers assessed each study and whether they worked independently, and if applicable, details of automation tools used in the process.	Line 78-84
Effect measures	12	Specify for each outcome the effect measure(s) (e.g. risk ratio, mean difference) used in the synthesis or presentation of results.	n/a
Synthesis	13a	Describe the processes used to decide which studies were eligible for each synthesis (e.g. tabulating the study intervention characteristics and	Line 86-91

Section and Topic	Item #	Checklist item	Location where item is reported
methods		comparing against the planned groups for each synthesis (item #5)).	
	13b	Describe any methods required to prepare the data for presentation or synthesis, such as handling of missing summary statistics, or data conversions.	Line 86-91
	13c	Describe any methods used to tabulate or visually display results of individual studies and syntheses.	Line 86-91
	13d	Describe any methods used to synthesize results and provide a rationale for the choice(s). If meta-analysis was performed, describe the model(s), method(s) to identify the presence and extent of statistical heterogeneity, and software package(s) used.	Line 86-91
	13e	Describe any methods used to explore possible causes of heterogeneity among study results (e.g. subgroup analysis, meta-regression).	n/a
	13f	Describe any sensitivity analyses conducted to assess robustness of the synthesized results.	n/a
Reporting bias assessment	14	Describe any methods used to assess risk of bias due to missing results in a synthesis (arising from reporting biases).	Line 79-84
Certainty assessment	15	Describe any methods used to assess certainty (or confidence) in the body of evidence for an outcome.	n/a
**RESULTS**			
Study selection	16a	Describe the results of the search and selection process, from the number of records identified in the search to the number of studies included in the review, ideally using a flow diagram.	Line 93-114
	16b	Cite studies that might appear to meet the inclusion criteria, but which were excluded, and explain why they were excluded.	Line 93-114
Study characteristics	17	Cite each included study and present its characteristics.	Line 145-374
Risk of bias in studies	18	Present assessments of risk of bias for each included study.	93-114
Results of individual studies	19	For all outcomes, present, for each study: (a) summary statistics for each group (where appropriate) and (b) an effect estimate and its precision (e.g. confidence/credible interval), ideally using structured tables or plots.	n/a
Results of syntheses	20a	For each synthesis, briefly summarise the characteristics and risk of bias among contributing studies.	Line 145-374
	20b	Present results of all statistical syntheses conducted. If meta-analysis was done, present for each the summary estimate and its precision (e.g. confidence/credible interval) and measures of statistical heterogeneity. If comparing groups, describe the direction of the effect.	n/a
	20c	Present results of all investigations of possible causes of heterogeneity among study results.	Line 145-374
	20d	Present results of all sensitivity analyses conducted to assess the robustness of the synthesized results.	n/a
Reporting biases	21	Present assessments of risk of bias due to missing results (arising from reporting biases) for each synthesis assessed.	n/a
Certainty of evidence	22	Present assessments of certainty (or confidence) in the body of evidence for each outcome assessed.	n/a
**DISCUSSION**			
Discussion	23a	Provide a general interpretation of the results in the context of other evidence.	Line 376-459

Section and Topic	Item #	Checklist item	Location where item is reported
	23b	Discuss any limitations of the evidence included in the review.	Line 376-459
	23c	Discuss any limitations of the review processes used.	Line 441-446
	23d	Discuss implications of the results for practice, policy, and future research.	Line 447-459
**OTHER INFORMATION**			
Registration and protocol	24a	Provide registration information for the review, including register name and registration number, or state that the review was not registered.	n/a
	24b	Indicate where the review protocol can be accessed, or state that a protocol was not prepared.	n/a
	24c	Describe and explain any amendments to information provided at registration or in the protocol.	n/a
Support	25	Describe sources of financial or non-financial support for the review, and the role of the funders or sponsors in the review.	Line 474
Competing interests	26	Declare any competing interests of review authors.	Line 476-478
Availability of data, code and other materials	27	Report which of the following are publicly available and where they can be found: template data collection forms; data extracted from included studies; data used for all analyses; analytic code; any other materials used in the review.	Line 460-468

For more information, visit: http://www.prisma-statement.org/.

Figure S1. Search strategy through electronic databases.

MEDLINE:

Search: antibiotic and covid-19

Filters: Clinical Study, from 2022 - 2024

(("anti-bacterial agents"[Pharmacological Action] OR "anti-bacterial agents"[MeSH Terms] OR ("anti-bacterial"[All Fields] AND "agents"[All Fields]) OR "anti-bacterial agents"[All Fields] OR "antibiotic"[All Fields] OR "antibiotics"[All Fields] OR "antibiotic s"[All Fields] OR "antibiotical"[All Fields]) AND ("covid 19"[All Fields] OR "covid 19"[MeSH Terms] OR "covid 19 vaccines"[All Fields] OR "covid 19 vaccines"[MeSH Terms] OR "covid 19 serotherapy"[All Fields] OR "covid 19 nucleic acid testing"[All Fields] OR "covid 19 nucleic acid testing"[MeSH Terms] OR "covid 19 serological testing"[All Fields] OR "covid 19 serological testing"[MeSH Terms] OR "covid 19 testing"[All Fields] OR "covid 19 testing"[MeSH Terms] OR "sars cov 2"[All Fields] OR "sars cov 2"[MeSH Terms] OR "severe acute respiratory syndrome coronavirus 2"[All Fields] OR "ncov"[All Fields] OR "2019 ncov"[All Fields] OR (("coronavirus"[MeSH Terms] OR "coronavirus"[All Fields] OR "cov"[All Fields]) AND 2019/11/01:3000/12/31[Date - Publication]))) AND ((clinicalstudy[Filter]) AND (2023:2024[pdat]))

SCOPUS:

antibiotic AND treatment AND covid-19 AND PUBYEAR > 2021 AND PUBYEAR < 2025 AND (LIMIT-TO (DOCTYPE, "ar”)) AND (LIMIT-TO (EXACTKEYWORD, "Hospital Patient”))

Table S3. Quality appraisal of the included studies.

**1. Was the sample frame appropriate to address the target population? Y/N**<br>A suitable sample frame is one that represents hospitalized COVID-19 patients, or that presents data from patients both with and without COVID-19 in a distinguishable manner.
**2. Were study participants recruited in an appropriate way? Y/N**<br>A proper recruitment hinged on non-random selection and on enrolment of any available patient with COVID-19 infection.
**3. Was the sample size adequate? Y/N**<br>A sample size was deemed as adequate if ≥100 patients. This arbitrary solution was ultimately considered a good compromise for the present analysis.
**4. Were the study subjects and setting described in detail? Y/N**<br>A detailed description relied on clear differentiation between the number of patients and episodes of bacterial infection, and on an adequate distinction between colonization and actual infections.
**5. Was data analysis conducted with sufficient coverage of the identified sample? Y/N**<br>Coverage was deemed sufficient if required data were available for all the period/s indicated in the study.
**6. Were valid methods used for the identification of the condition? Y/N**<br>The gold standard method to diagnose bacterial infection is based on culture-dependent antibiotic sensitivity testing results after a confirmed clinical diagnosis of bacterial infection.
**7. Was the condition measured in a standard, reliable way for all participants? Y/N**<br>The criterium was not meet if data from a given study included bacterial colonisation that could not be separated from true bacterial infection episodes
**8. Was the response rate adequate, and if not, was the low response rate managed appropriately? Y/N**<br>Response rate was considered adequate if the species of bacteria was ultimately identified for all isolates from bacterial infection episodes
**9. Was there appropriate statistical analysis? NA**<br>Proper statistical analysis to calculate prevalence of bacterial infection. Point-prevalence was considered appropriately calculated when the sum of all patients with a diagnosis of bacterial infection was divided by the study population.

Y: yes; N: no; NA: not applicable.

Table S4. Summary description of the studies on the prevalence of bacterial infection and antibiotics use in hospitalized COVID-19 patients included in the systematic review.

Author, Year and Country	Study Population	Study Design	Study Aim	Setting	Methods	Study Results
			**Wards**			
Ng TM et al. 2022, Singapore [1] High quality (8)	717 hospitalized COVID-19 patients	Observational cohort study	To evaluate the frequency of bacterial co-infection and the prevalence of antibiotic use among hospitalized COVID-19 patients	1 tertiary hospital, from January to April 2020	Data were extracted from the hospital's medical records. Medical records were screened for positive microbiology results and concomitant suspected or confirmed bacterial co-infections within 5 days of COVID-19 diagnosis	Of 717 patients included, 86 (12.0%) were treated with antibiotics and 26 (3.6%) had a documented bacterial infection. Antibiotic treatment was not independently associated with lower 30-day or in-hospital mortality rates
Milas S et al. 2022, Belgium [2] Medium quality (6)	164 COVID-19, hospitalized patients	Retrospective study	To determine the risk factors associated with antibiotic use and to assess the impact of antimicrobial therapy on the length of the hospital stay, secondary intensive care unit admission and in-hospital mortality	A tertiary hospital located with 1374 beds (including 44 intensive care beds) Between 11 March 2020, and 3 May 2020	Data were collected from the electronic records on demographics, comorbidities, COVID-19 disease severity, laboratory test results, radiological examination results, treatments, length of hospital stay, outcomes	100/164 patients (61%) received antibiotic treatment. 28/164 (17.1%) had a confirmed infection, mostly of the urinary tract (18/28, 64.3%). Factors associated with antibiotic use: being hospitalized in the intensive care (OR: 4.59; 95% CI: 1.07–19.71), age > 65 years (OR: 4.16; 95% CI: 1.72–10.05), arrival from a nursing home (OR: 4.59; 95% CI: 1.11–19.71), diabetes (OR: 4.35; 95% CI: 1.26–14.93), bilateral consolidation (OR: 9.92; 95% CI: 2.40–41.06) and a C-reactive protein level >60

						mg/L (OR: 2.46; 95% CI: 1.13–5.37)
Rebold N et al. 2022, US [3] Medium quality (7)	595 hospitalized COVID-19 patients	Retrospective cohort study	To describe factors associated with a bacterial bloodstream coinfection in COVID-19 patients	4 hospitals. Observation period of 4 weeks, between March 8, 2020, and April 4, 2020	Variables of interest were compared between true bloodstream infection (n = 25) and all other COVID-19 cases (n = 570)	True bloodstream coinfection occurred in 4.2% (25/595) of the COVID-19 patients. Neurological symptoms were significantly higher in the bloodstream infection group (OR: 3.27, *p*<0.01). Thirty-day mortality was higher among true bloodstream infection (*p*<0.01)
Ahava MJ et al. 2023, Finland [4] Medium quality (6)	585 laboratory-confirmed patients with COVID-19	Retrospective, observational study	To evaluate the incidence, aetiology and outcome of blood and respiratory tract bacterial infections among hospitalized COVID-19 patients	Hospital district. between 27 February and 21 June 2020 were	Blood and respiratory tract culture reports of hospitalized patients were collected and analysed for their association with 90-day case-fatality using multivariable regression analysis	A bacterial infection was diagnosed in 40/585 (6.8%) patients with COVID-19. Gram-negative bacteria were the most common in respiratory samples, whilst Gram-positive bacteria predominated in blood cultures. Patients with bacterial infection had longer hospital stay compared to patients without bacterial infection (31 versus 9 days; *p*<0.001)
Gajic I et al. 2023, Serbia [5] High quality (8)	7249 hospitalized COVID-19 patients	Retrospective, observational study	To evaluate the aetiology of bacterial infections, antibiotic resistance patterns, treatment approaches,	3 regional hospitals in Serbia, from 1st January 2021 to 16th February 2022	Data were extracted from the hospital's medical records. The isolated bacteria were identified using "VITEK2" system, analytical profile index, and MALDI-TOF mass	The prevalence of bacterial infections was 12.9%, most of them being hospital-acquired (11.5%). Bloodstream (37.7%) and respiratory tract infections (25.6%) were the most common bacterial infections.

			comorbidities, risk factors, and mortality rate of hospitalized COVID-19 patients		spectrometry. Antimicrobial susceptibilities of bacteria were determined by the disk diffusion method, gradient test, VITEK2 system or broth microdilution test	The overall in-hospital mortality rate of COVID-19 patients with bacterial infections was 51.6%, while 91.7% of patients who required invasive mechanical ventilation died
Martin AJ et al. 2023, US [6] Medium quality (6)	198 adult, symptomatic, hospitalized COVID-19 Patients 18.	Multi-center, retrospective, observational study	To assess antibiotic prescribing trends and overall antibiotic use	3 hospitals within the University of Rochester Medical Center. From March 1, 2020, to May 31, 2020	Patients were identified from the electronic health record. The primary endpoint was the rate of antibiotic use in patients admitted for COVID-19	83% of patients received at least 1 course of antibiotics, with low rates of microbiologically confirmed bacterial co-infection
Desai K et al. 2022, Us [7] Medium quality (7)	About 13 million patients with a combined total of approximately 2.4 million antibiotic prescriptions	Retrospective observational cohort study	To compare antibiotic prescribing trends among COVID-19 patients categorized by their disease severity and to compare these trends to the counterpart non-COVID-19 patient during the pandemic	Large hospital network. January-November 2020	Data were collected from healthcare administrative databases. Cohorts were categorized based on diagnosis codes	Overall, in the analytical sample of 13 million patients, 19% received an antibiotic prescription within 7 days of the first diagnosis of COVID-19. Within the COVID-19 positive cohorts, about 11% received an antibiotic prescription, while the non-COVID-19 cohorts, about 19.7% received an antibiotic. Among patients with antibiotic prescriptions, about 22.6% received an “inappropriate” antibiotic. Among patients prescribed antibiotics, azithromycin was the

						most common, ranging from 21.8 to 44.8% for each cohort
Di Lorenzo A et al. 2023, Italy [8] Medium quality (7)	482 COVID-19 patients	Single-center, retrospective, observational study	To describe antibiotic prescription practice in COVID-19 patients before hospitalization and bacterial and viral co-infection rates	1 University hospital in Italy, from 1 January 2021 to 31 December 2021	Data were extracted from the hospital electronic record. Patients were stratified into 5 groups according to the maximal oxygen supply/ventilation support required during the hospitalization	In total, 151 patients (31.3%) received home antibiotics without any association with the outcome. 3,5% (15/428) patients were positive for *S. pneumoniae*
Habib G et al. 2022, Pakistan [9] Medium quality (6)	3492 COVID-19 hospitalized patients	Observational, retrospective	To assess the pathophysiology of methicillin-resistant *S. aureus* superinfection in COVID-19 patients	2 hospitals in Pakistan, between the years 2020 and 2021	Screening of bacterial isolates from COVID-19 patients. The prevalence of MRSA was calculated from the day of admission to 25 days of hospitalization. Nasopharyngeal and endotracheal aspirate specimens were collected from patients and were transferred to a bacteriological laboratory	Among the 3492 included patients, 224 (6.4%) MRSA strains were isolated. The prevalence of MRSA was 7.33% in patients aged ≥50 years, 5.1% in patients aged <50. Bacterial pneumonia increased the mortality rate from 2.3% to 25.23%
Widere JC et al. 2023, US [10] High quality (8)	322,867 patients hospitalized with COVID-19	Retrospective propensity-matched cohort study using the National COVID Cohort Collaborative database	To investigate temporal trends and outcomes associated with early antibiotic prescribing in patients hospitalized with COVID-19	66 health systems throughout the US. Between March 2020 and June 2022	Data were extracted from the participating hospital electronic records. Patients were defined to have early antibiotic use if they received at least 3 calendar days of intravenous antibiotics	Among the 322,867 hospitalizations, 43,089 patients received early antibiotics (13,3%). Patients who received early antibiotics were more likely to be older, male, obese, current smokers, and have more comorbid conditions. Average rates of

					within the first 5 days of admission	early antibiotics varied significantly between centers and over time. Among nonsurvivors, the median length of stay before mortality was 11 days (interquartile range 5–19 days). Ceftriaxone and azithromycin were the most prescribed antibiotics
Subagdja MFM et al. 2022, Indonesia [11] High quality (8)	2786 adult COVID-19 patients	Retrospective cross-sectional study	To describe the antibiotic resistance in COVID-19 patients with culture-proven bacterial infection	1 hospital. From March 2020 to October 2021	Laboratory-based surveillance approach: data were obtained from the hospital information system and merged with the culture and antibiotic susceptibility test from laboratory information system	The prevalence of bacterial infection among COVID-19 patients was 16.4%, predominating Gram-negative bacteria. High range resistance to ampicillin-sulbactam (24–100%), ceftriaxone (22–81%), cefotaxime (22–73%) and ciprofloxacin (20–86%) were reported among the Gram-negative bacteria
Bilan J et al. 2022, UK [12] High quality (8)	266 older patients with COVID-19	Single centre, observational cohort study	To investigate the occurrence and outcomes from possible superadded infections within 2 weeks of hospitalization in older adults with COVID-19	1 hospital older adults ward, between 1st October and 1st December 2020	The primary outcome was inpatient death occurring within 90 days of COVID-19 diagnosis. The secondary outcome was length of stay in hospital. Associations were described using univariable and multivariable models, and time to event data	115/266 (43%) patients had evidence of superadded infections. Patients with superadded infections were more likely to die (45.2 versus 30.7%, *p*: 0.02) and had an increased length of stay (23 versus 18 days, *p*: 0.026)

Maki KR et al. 2022, US [13] High quality (8)	358 hospitalized cancer patients ≥18 years with COVID-19	Retrospective cohort study	To characterize bacterial infections and antibiotic utilization in hospitalized cancer patients with COVID-19	Tertiary cancer center, between March 1, 2020, and May 31, 2020	Patients were classified with mild, moderate or severe COVID-19. The primary outcome was bacterial infection rate within 30 days of COVID-19 onset. Secondary outcomes included the proportion of patients receiving antibiotics and antibiotic length of therapy	Of 358 patients, 234 (65%) had a solid tumor. The proportion of patients with bacterial infection increased with COVID-19 severity: mild (n: 47, 35%) versus moderate (n: 49, 51%) versus severe (n: 104, 81%) (*p*<0.0001). 274 patients (77%) received antibiotics for a median of 4 days. The median antibiotic antibiotic length of therapy were 7 days with 1 infection and 20 days with multiple infections (*p*<0.0001). Antibiotic durations were 1 day for patients with mild COVID-19, 4 days for patients with moderate COVID-19, and 8 days for patients with severe COVID-19 (*p*<0.0001)
**Intensive care**						
Gragueb-Chatti I et al. 2022, France [14] Medium quality (7)	398 patients admitted for a documented COVID-19 and requiring mechanical ventilation for ≥48 h	Observational, retrospective study	To assess the incidence, outcomes and risk factors of ventilator associated pneumonia recurrences	3 intensive care units, from March 2020 to May 2021	Main outcome was the incidence of ventilator associated pneumonia recurrences. Secondary outcomes were the duration of mechanical ventilation, hospital length of stay and mortality	A total of 236 (59%) patients had at least one ventilator associated pneumonia episode and 109 (46%) of these patients developed at least one recurrence. The incidence of ventilator associated pneumonia recurrence was 29.6%.

						Patients with a ventilator associated pneumonia recurrence had a longer intensive care unit length of stay (46 versus 22 days; *p*<0.001). The 90-day mortality was higher in the recurrence group as compared with the no ventilator associated pneumonia group only (31.2 versus 21.0% (*p*: 0.021)
Aissaoui Y et al. 2022, Morocco [15] Medium quality (6)	155 severe COVID-19 patients admitted to the intensive care unit. The median age was 68 years	Retrospective study	To determine the prevalence of bacterial pulmonary co-infections and superinfections in severe COVID-19 pneumonia, the micro-organisms involved, and the impact of these infections on survival	Moroccan Intensive care unit, between April 2020 and April 2021	The diagnosis of pulmonary co-infections and superinfections was based on the identification of pathogens from lower respiratory tract samples. Co-infection was defined as the identification of a respiratory pathogen, diagnosed concurrently with SARS-CoV-2 pneumonia	A large proportion of patients (68%) received antibiotics before intensive care unit admission. The prevalence of co-infections, healthcare associated pneumonia and ventilator-associated pneumonia was respectively 4%, 12% and 40% (64 ventilator associated pneumonia/1000 ventilation days). The proportion of extra-drug resistant bacteria was 78% for *Acinetobacter spp.* and 24% for *Enterobacterales*. Overall intensive care unit mortality in this cohort was 64.5%
Sysiak-Sławecka J et al. 2023, Poland [16]	29 critically ill patients requiring mechanical ventilation	Observational cohort study	To assess the prevalence of superinfection, mortality, length of stay,	University hospital's intensive care unit	15 patients who required venovenous extracorporeal membrane support were compared to a	No difference in the number of superinfections and in mortality between the two study groups. The mortality

Medium quality (6)	due to COVID-19		antibiotics used during treatment, and the impact of immunomodulatory drugs on secondary infections		control group of 14 individuals without extracorporeal support	rate was 81% in patients with superinfection versus 25% in those without co-infection (*p*: 0.009)
Russo A et al. 2023, Italy [17] Medium quality (7)	73 COVID-19 patients admitted in intensive care unit and developing carbapenem-resistant A. baumannii infections	Single-centre, retrospective	To evaluate risk factors associated with survival or death in the COVID-19 intensive care unit at 30 days from ventilatory associated pneumonia onset	Italian hospital. From March 2020 to August 2022	Patient data were collected from medical charts and from computerized hospital databases or clinical charts using a pre-established questionnaire. The inclusion criteria were: (1) age ≥18 years; (2) blood culture and respiratory tract culture positive for carbapenem-resistant A. baumannii; and (3) clinical signs and symptoms consistent with ventilatory associated pneumonia	Overall, 59 patients (80.8%) died at 30 days. 54 (74%) patients were treated with a colistin-containing regimen and 19 (26%) were treated with a cefiderocol-containing regimen. Chronic obstructive pulmonary disease and age were independently associated with 30-day mortality. Conversely, cefiderocol-containing regimens and cefiderocol plus fosfomycin in combination were independently associated with 30-day survival
Petrakis V et al. 2023, Greece [18] Medium quality (6)	4569 bacterial strains isolated from COVID-19 hospitalized patients	Retrospective study	To evaluate the incidence of antimicrobial resistance and the management of bloodstream infections before and during the	1 university hospital. From January 2018 to December 2022	Data were collected from the microbiology laboratory per semester regarding the isolated strains of Gram-positive and Gram-negative bacteria in blood cultures and respiratory samples. Additionally, bloodstream	An increasing trend was reported compared to the pre-pandemic period in the incidence of resistant Gram-negative bacteria. Prior antimicrobial use and the rate of hospital-acquired infections were increased significantly during the pandemic. Detection of the source of

			COVID-19 pandemic		infections with requested infectious disease consultations were reported	infection and timely administration of appropriate antimicrobial agents were more frequently recorded before the pandemic. 28-day mortality was significantly reduced in cases with bedside consultations

CI: condidence interval; OR: odds ratio; MRSA: Methicillin-Resistant *Staphylococcus Aureus*; MALDI: matrix assisted laser desorption-ionisation time of flight.

Table S5. Summary description of the studies on the efficacy of early antibiotic administration in COVID-19 patients.

Author, Year and Country	Study Population	Study Design	Study Aim	Setting	Methods	Study Results
Dhar R et al. 2023, India and UK [19] Low quality (5)	387 COVID-19, hospitalized patients aged 40–90 years	Randomized, controlled, open-label, non-blinded parallel group trial	To test the hypothesis that doxycycline is effective in preventing intensive care unit admission in hospitalized COVID-19 patients	6 hospitals in India	Patients were randomised 1:1 to receive standard of care plus doxycycline (n: 192) or standard of care only (n: 195). The primary outcome was the need for intensive care unit admission	Among all 387 participants, 77 (19.9%) needed admission at the intensive care unit. Doxycycline was associated with a relative risk reduction for intensive care unit admission (31.6%)
Yin X et al. 2022, China [20] Medium quality (6)	1373 inpatients with non-severe COVID-19, admitted wthout bacteral infection	Multi-centre retrospective cohort study	To evaluate the effect of early antibiotic use in patients with non-severe COVID-19 admitted without bacterial infection	4 hospitals in China, from 31 December 2019 to 31 March 2020	3 physicians involved in the treatment of COVID-19 performed a chart review of the electronic medical records of patients and determined bacterial infection using the majority rule. Patients were divided into two groups according to their exposure	During the 30-day follow-up period, the proportion of patients who progressed to severe COVID-19 in the early antibiotic use group was almost 1.4 times that of the comparison group. In the mixed-effect model, the early use of antibiotics was associated with higher probability of developing severe COVID-

					to antibiotics within 48 h of admission. The outcomes were progression to severe COVID-19, length of stay >15 days and mortality rate	19 and staying in hospital for >15 days. However, there was no significant association between early use of antibiotics and mortality. In subgroup analysis, azithromycin did not improve disease progression and length of stay
Bergami M et al. 2023, Croatia, Italy, Macedonia, Romania, and Serbia [21] High quality (8)	4462 patients with COVID-19, non-vaccinated for SARS-CoV-2	Retrospective cohort study	To examine the benefits and risks associated with azithromycin use within 24 h from hospital admission compared with standard of care	7 medical centers of 5 European countries. From December 2021 to February 2022	Patients were scheduled to receive azithromycin 500 mg by mouth or intravenous injection once a day for 10 days. The primary outcome measure was all cause mortality within 30 days of hospital admission	Azithromycin therapy was consistently associated with an increased risk of acute heart failure in patients with preexisting cardiovascular disease (risk ratio: 1.48 (95% CI: 1.06–2.06)
Biagioni E et al. 2023, Italy [22] Medium quality (6)	348 adult patients requiring invasive mechanical ventilation for severe respiratory failure related to SARS-CoV-2	Observational, pre-post study	To evaluate whether the introduction of selective digestive decontamination is effective in reducing the occurrence of ventilator-associated pneumonia in COVID-19 patients	3 COVID-19 intensive care units in an Italian hospital from 22 February 2020 to 8 March 2022	Selective digestive decontamination consisted of a tobramycin sulfate, colistin sulfate, and amphotericin B suspension applied in the patient's oropharynx and the stomach via a nasogastric tube	In the 86 patients (32.9%) who received selective digestive decontamination, the occurrence of ventilator-associated pneumonia decreased by 7.7%. The use of selective digestive decontamination reduced the occurrence of ventilator-associated pneumonia (HR: 0.536, CI: 0.338–0.851; p: 0.017)

CI: condidence interval; HR: hazard ratio.

Table S6. Summary description of the studies on the use of sepsis biomarkers to improve antibiotic use in COVID-19 hospitalized patients.

Author, Year and Country	Study Population	Study Design	Study Aim	Setting	Methods	Study Results
Conlon ASC et al. 2022, US [23] High quality (8)	793 COVID-19 hospitalized patients	Retrospective, observational study	To describe the natural course of procalcitonin in COVID-19 patients and the correlation between procalcitonin and antimicrobial prescribing to provide insight into best practices for procalcitonin data utilization in antimicrobial stewardship in the COVID-19 population	1 hospital, between March 2020 and October 2021	Data were extracted from the hospital electronic record to identify patients with bacterial pneumonia and antibiotic use. Multivariable models were used to assess associations of procalcitonin level and bacterial pneumonia with antimicrobial use	Of 793 patients, 224 (28.2%) were initiated on antibiotics: 33 (14.7%) had proven or probable bacterial pneumonia, 125 (55.8%) had possible bacterial pneumonia, and 66 (29.5%) had no bacterial pneumonia. Initial procalcitonin level was highest for those with proven/probable bacterial pneumonia and was associated with antibiotic initiation. Initial procalcitonin, change in procalcitonin over time and bacterial pneumonia were associated with antibiotic duration
Hessels LM et al. 2023, The Netherlands [24] High quality (8)	1,335 COVID-19 patients	Multicenter cohort study	To determine if the use of a procalcitonin-guided antibiotic protocol safely reduce the use of antibiotics in COVID-19 patients	A large teaching hospital. between October 2020 and July 2021	At procalcitonin levels <0.25, antibiotics were discouraged; between 0.25 and 0.5 antibiotics could be considered; >0.5, antibiotics were recommended. 3 groups of patients with COVID-19 were compared in terms of antibiotic consumption. 1 group treated based on a procalcitonin -algorithm in 1	Antibiotic prescription during the first 7 days was 26.8% in the procalcitonin group, 43.9% in the non- procalcitonin group in the same hospital, and 44.7% in the non- procalcitonin group in other hospitals. Patients in the procalcitonin group had lower odds of receiving antibiotics in the first 7 days of admission (OR: 0.33; 95% CI: 0.16–0.66). The proportion of

					hospital (n: 216) and 2 control groups without procalcitonin measurements	patients receiving antibiotic prescription during the total admission was 35.2%, 43.9%, and 54.5%, respectively
Fartoukh M et al. 2023, France [25] Medium quality (7)	194 adult, critically ill COVID-19 patients	Multicentre, parallel-group, open-label, randomized controlled trial	13 intensive care units in France. Between April and November 2020	To assess the efficacy and safety on antibiotic exposure of a strategy combining a respiratory multiplex PCR panel and daily procalcitonin	Patients were assigned (1:1) to the control strategy, in which antibiotic streamlining remained at the discretion of the physicians, or interventional strategy, consisting of using multiplex PCR and daily procalcitonin measurements within the first 7 days of randomization to streamline initial antibiotic therapy, with antibiotic continuation encouraged when procalcitonin was >1 ng/mL and discouraged if <1 ng/mL or decreased by 80% from baseline	191 patients were retained in the intention-to-treat analysis. Respiratory bacterial co-infection was detected in 48.4% (45/93) and 21.4% (21/98) in the interventional and control group, respectively. The number of antibiotic-free days was 12 (0.0; 25.0) and 14 (0.0; 24.0) days, respectively (difference, -2.0, (95% CI: -10.6 to 6.6), p: 0.89). Superinfection rates were 51.6% and 48.5%, respectively. Mortality rates and intensive care units' lengths of stay did not differ between groups
Sathitakorn O et al. 2023, Thailand [26] Medium quality (7)	406 hospitalized COVID-19 patients	Quasi-experimental multicenter study	2 tertiary care hospitals, From March 1, 2020 to February 28, 2022	To evaluate the role of procalcitonin and the "Clinical Pulmonary for Infection" score in reducing	1-year pre-implementation period of the protocol and a 1-year post-implementation period. During period 1, no antibiotic protocols for COVID-19 patients were available	Compared to period 1, the overall inappropriate antibiotic use was significantly reduced during period 2: 63.5% versus 31.3% of (p<0.01). Overall, inappropriate use was significantly reduced among severe

				inappropriate antibiotic use and incidence of multidrug-resistant organisms among COVID-19 patients	at either hospital. During period 2, upon admission, the researchers calculated the "CPIS" score and ordered admission procalcitonin for all COVID-19 patients. For those with a score <6 and a procalcitonin <0.5 μg/L, no antibiotics were initiated. Procalcitonin-CPIS score were reassessed on day 3 and if score was <6 and procalcitonin was <0.5 μg/L or <80% from admission level antibiotics were discontinued	COVID-19 patients (80.8% versus 39.1%, $p<$ 0.01), but it was not reduced among mild patients (8.7% versus 7.5%, p: 0.83). In period 2, there was a significantly lower incidence of MDR organisms (p: 0.04). The protocol was associated with a significantly shorter total antibiotic duration (7 versus 0 days. $p<0.01$) and length of stay (13 versus 10 days, $p<0.01$). The 30-day mortality was not significantly different

CI: confidence interval; OR: odds ratio, MDR: multidrug-resistant; PCR: polymerase chain reaction.

Table S7. Summary description of the studies on the efficacy of antimicrobial stewardship programs in COVID-19 Hospitalized Patients.

Author, Year and Country	Study Population	Study Design	Study Aim	Setting	Methods	Study Results
Sibani M et al. 2023, Italy [27] Medium quality (7)	1743 patients	Controlled, before-after study	4 units in a 1350-bed tertiary care, university hospital, from March 2020 to May 2021	To evaluate the impact of a multiphase and customized antimicrobial stewardship intervention in COVID-19 wards	During the first wave, COVID-19 forced the complete withdrawal of hospital antibiotic stewardship. In the second wave, antibiotic guidance calibration for COVID-19 patients was implemented in all units, with enhanced	In the first wave, the overall normalized days-of-therapy in units 2–4 significantly exceeded the 2019 level ($p<0.05$). After the introduction of antimicrobial stewardship activities, consumption decreased in the intervention units to a significantly lower level when compared to 2019 ($p<0.05$). Antimicrobial

					stewardship activities in Units 1, 2, and 3 (intervention units). Antimicrobial usage during the 3 waves of the COVID-19 pandemic was compared to the 12-month prepandemic unit (Unit 4 acted as the control)	stewardship activities resulted in a decreased amount of total antibiotic consumption over time and positively affected the watch class and piperacillin-tazobactam use in the involved units
Spernovasilis N et al. 2023, Greece [28] Medium quality (6)	1268 patients hospitalized during the COVID-19 pandemic who received carbapenems for at least 24 h during the 24 month study period	Retrospective-prospective, quasi-experimental, before–after study	A tertiary hospital	To measure the impact of a carbapenem-focused antimicrobial stewardship programme on the antibiotic consumption and patient outcomes	A multifaceted antimicrobial stewardship intervention was implemented: The infectious diseases specialist was alerted by the pharmacy upon prescription order for a carbapenem and provided unsolicited in-person consultation within 72 h. Unsolicited follow-up bedside consultation was provided daily or every other day	The proportion of admitted patients who received carbapenems decreased from 4.1% to 2.3%, $p<0.001$. 30-day mortality following initiation of carbapenem treatment and all-cause in-hospital mortality remained unaltered. In the post-implementation period, acceptance of the antimicrobial stewardship intervention was associated with lower odds of 30-day mortality (OR: 0.36; 95% CI: 0.18–0.70) and higher rate of treatment success (HR: 2.45; 95% CI: 1.59–3.77)
Giannella M et al. 2022, Italy [29] High quality (8)	1733 COVID-19 patients were analyzed.	Multicenter observational study	3 large Italian hospital. From December, 2020 through February 2022	To build a predictive model able to stratify the risk of bacterial co-	Endpoint was microbiologically documented bacterial co-infection diagnosed within 72 h from	Empirical antibiotics were started in 64.2 and 59.5% of patients with and without co-infection (p: 0.35). At multivariable analysis in the derivation cohort:

Median age 69 years		infection at hospitalization in patients with COVID-19	hospitalization. The cohort was randomly split into derivation and validation cohort	white blood cells ≥7.7/mm^3, Procalcitonin ≥0.2 ng/mL and Charlson index ≥5 were risk factors for bacterial co-infection. The predictive model showed positive predictive value 16.0% and negative predictive value 97.5%

CI: confidence interval; OR: odds ratio; HR: hazard ratio.

References

1. Ng, T.M.; Ong, S.W.X.; Loo, A.Y.X.; Tan, S.H.; Tay, H.L.; Yap, M.Y.; Lye, D.C.; Lee, T.H.; Young, B.E. Antibiotic Therapy in the Treatment of COVID-19 Pneumonia: Who and When? *Antibiotics* **2022**, *11*, 184. https://doi.org/10.3390/antibiotics11020184.
2. Milas, S.; Poncelet, A.; Buttafuoco, F.; Pardo, A.; Lali, S.E.; Cherifi, S. Antibiotic use in patients with Coronavirus disease 2019 (COVID-19): Outcomes and associated factors. *Acta Clin. Belg. Int. J. Clin. Lab. Med.* **2022**, *77*, 579–587.
3. Rebold, N.; Alosaimy, S.; Morrisette, T.; Holger, D.; Lagnf, A.M.; Ansari, I.; Belza, A.C.; Cheaney, L.; Hussain, H.; Herbin, S.R.; et al. Clinical Characteristics Associated with Bacterial Bloodstream Coinfection in COVID-19. *Infect. Dis. Ther.* **2022**, *11*, 1281–1296.
4. Ahava, M.J.; Kortela, E.; Forsblom, E.; Pätäri-Sampo, A.; Friberg, N.; Meretoja, A.; Kivivuori, S.-M.; Lappalainen, M.; Kurkela, S.; Järvinen, A.; et al. Low incidence of severe bacterial infections in hospitalised patients with COVID-19: A population-based registry study. *Infect. Dis.* **2023**, *55*, 132–141.
5. Gajic, I.; Jovicevic, M.; Popadic, V.; Trudic, A.; Kabic, J.; Kekic, D.; Ilic, A.; Klasnja, S.; Hadnadjev, M.; Popadic, D.J.; et al. The emergence of multi-drug-resistant bacteria causing healthcare-associated infections in COVID-19 patients: A retrospective multi-centre study. *J. Hosp. Infect.* **2023**, *137*, 1–7. https://doi.org/10.1016/j.jhin.2023.04.013.
6. Martin, A.J.; Shulder, S.; Dobrzynski, D.; Quartuccio, K.; Pillinger, K.E. Antibiotic Use and Associated Risk Factors for Antibiotic Prescribing in COVID-19 Hospitalized Patients. *J. Pharm. Pract.* **2023**, *36*, 256–263. https://doi.org/10.1177/08971900211030248.
7. Desai, K.; Arora, P.; Ghanekar, S.; Johnson, K.; Harris, I. Antibiotic prescribing trends in the US during the first 11 months of the COVID-19 pandemic. *Res. Social Adm. Pharm.* **2022**, *18*, 3855–3859. https://doi.org/10.1016/j.sapharm.2022.05.008.
8. Di Lorenzo, A.; Campogiani, L.; Iannetta, M.; Iannazzo, R.; Imeneo, A.; Alessio, G.; D'Aquila, V.; Massa, B.; Fato, I.; Rindi, L.V.; et al. The Impact of Viral and Bacterial Co-Infections and Home Antibiotic Treatment in SARS-CoV-2 Hospitalized Patients at the Policlinico Tor Vergata Hospital, Rome, Italy. *Antibiotics* **2023**, *12*, 1348. https://doi.org/10.3390/antibiotics12091348.
9. Habib, G.; Mahmood, K.; Gul, H.; Tariq, M.; Ain, Q.U.; Hayat, A.; Rehman, M.U. Pathophysiology of Methicillin-Resistant Staphylococcus aureus Superinfection in COVID-19 Patients. *Pathophysiology* **2022**, *29*, 405–413.
10. Widere, J.C.; Davis, C.L.; Loomba, J.J.; Bell, T.D.; Enfield, K.B.; Barros, A.J.; N3C Consortium. Early Empiric Antibiotic Use in Patients Hospitalized With COVID-19: A Retrospective Cohort Study. *Crit. Care Med.* **2023**, *51*, 1168–1176. https://doi.org/10.1097/CCM.0000000000005901.
11. Subagdja, M.F.M.; Sugianli, A.K.; Prodjosoewojo, S.; Hartantri, Y.; Parwati, I. Antibiotic Resistance in COVID-19 with Bacterial Infection: Laboratory-Based Surveillance Study at Single Tertiary Hospital in Indonesia. *Infect. Drug Resist.* **2022**, *15*, 5849–5856.
12. Bilan, J.; Aggrey, K.; Quinn, T.J.; Lumsden, J.; Colquhoun, K. Occurrence and outcomes of possible superadded infections in older adults with COVID-19—Cohort study. *Eur. Geriatr. Med.* **2022**, *13*, 1161–1167.
13. Maki, K.R.; Steiger, S.N.; Su, Y.; Boumiza, A.; Tan, C.A.; Kerpelev, M.; Seo, S.K.; Cohen, N. Bacterial infections and antibiotic utilization varies by coronavirus disease 19 (COVID-19) severity in hospitalized cancer patients: Analysis from the first phase of the pandemic. *Infect Control. Hosp. Epidemiol.* **2023**, *44*, 413–419. https://doi.org/10.1017/ice.2022.129.
14. Gragueb-Chatti, I.; Hyvernat, H.; Leone, M.; Agard, G.; Peres, N.; Guervilly, C.; Boucekine, M.; Hamidi, D.; Papazian, L.; Dellamonica, J.; et al. Incidence, Outcomes and Risk Factors of Recurrent Ventilator Associated Pneumonia in COVID-19 Patients: A Retrospective Multicenter Study. *J. Clin. Med.* **2022**, *11*, 7097. https://doi.org/10.3390/jcm11237097.

15. Aissaoui, Y.; Ennassimi, Y.; Myatt, I.; El Bouhiaoui, M.; Nabil, M.; Bahi, M.; Arsalane, L.; Miloudi, M.; Belhadj, A. What happened during COVID-19 in African ICUs? An observational study of pulmonary co-infections, superinfections, and mortality in Morocco. *PLoS ONE* **2022**, *17*, e0278175. https://doi.org/10.1371/journal.pone.0278175.
16. Sysiak-Sławecka, J.; Wichowska, O.; Piwowarczyk, P.; Borys, M. The impact of bacterial superinfections on the outcome of critically ill patients with COVID-19 associated acute respiratory distress syndrome (ARDS)—a single-centre, observational cohort study. *Anaesthesiol. Intensiv. Ther.* **2023**, *55*, 163–167. https://doi.org/10.5114/ait.2023.130833.
17. Russo, A.; Bruni, A.; Gullì, S.; Borrazzo, C.; Quirino, A.; Lionello, R.; Serapide, F.; Garofalo, E.; Serraino, R.; Romeo, F.; et al. Efficacy of cefiderocol- vs colistin-containing regimen for treatment of bacteraemic ventilator-associated pneumonia caused by carbapenem-resistant Acinetobacter baumannii in patients with COVID-19. *Int. J. Antimicrob. Agents* **2023**, *62*, 106825. https://doi.org/10.1016/j.ijantimicag.2023.106825.
18. Petrakis, V.; Panopoulou, M.; Rafailidis, P.; Lemonakis, N.; Lazaridis, G.; Terzi, I.; Papazoglou, D.; Panagopoulos, P. The Impact of the COVID-19 Pandemic on Antimicrobial Resistance and Management of Bloodstream Infections. *Pathogens* **2023**, *12*, 780. https://doi.org/10.3390/pathogens12060780.
19. Dhar, R.; Kirkpatrick, J.; Gilbert, L.; Khanna, A.; Modi, M.M.; Chawla, R.K.; Dalal, S.; Maturu, V.N.; Stern, M.; Keppler, O.T.; et al. Doxycycline for the prevention of progression of COVID-19 to severe disease requiring intensive care unit (ICU) admission: A randomized, controlled, open-label, parallel group trial (DOXPREVENT.ICU). *PLoS ONE* **2023**, *18*, e0280745. https://doi.org/10.1371/journal.pone.0280745.
20. Yin, X.; Xu, X.; Li, H.; Jiang, N.; Wang, J.; Lu, Z.; Xiong, N.; Gong, Y. Evaluation of early antibiotic use in patients with non-severe COVID-19 without bacterial infection. *Int. J. Antimicrob. Agents.* **2022**, *59*, 106462. https://doi.org/10.1016/j.ijantimicag.2021.106462.
21. Bergami, M.; Manfrini, O.; Nava, S.; Caramori, G.; Yoon, J.; Badimon, L.; Cenko, E.; David, A.; Demiri, I.; Dorobantu, M.; et al. Relationship Between Azithromycin and Cardiovascular Outcomes in Unvaccinated Patients With COVID-19 and Preexisting Cardiovascular Disease. *J. Am. Heart Assoc.* **2023**, *12*, e028939. https://doi.org/10.1161/JAHA.122.028939.
22. Biagioni, E.; Ferrari, E.; Gatto, I.; Serio, L.; Farinelli, C.; Coloretti, I.; Talamonti, M.; Tosi, M.; Meschiari, M.; Tonelli, R.; et al. Role of Selective Digestive Decontamination in the Prevention of Ventilator-Associated Pneumonia in COVID-19 Patients: A Pre-Post Observational Study. *J. Clin. Med.* **2023**, *12*, 1432. https://doi.org/10.3390/jcm12041432.
23. Conlon, A.S.C.; Chopra, Z.; Cahalan, S.; Cinti, S.; Rao, K. Effects of procalcitonin on antimicrobial treatment decisions in patients with coronavirus disease 2019 (COVID-19). *Infect. Control. Hosp. Epidemiol.* **2023**, *44*, 1314–1320. https://doi.org/10.1017/ice.2022.262.
24. Hessels, L.M.; Speksnijder, E.; Paternotte, N.; van Huisstede, A.; Thijs, W.; Scheer, M.; van der Steen-Dieperink, M.; Knarren, L.; van Den Bergh, J.P.; Winckers, K.; et al. Procalcitonin-Guided Antibiotic Prescription in Patients With COVID-19: A Multicenter Observational Cohort Study. *Chest* **2023**, *164*, 596–605. https://doi.org/10.1016/j.chest.2023.04.032.
25. Fartoukh, M.; Nseir, S.; Mégarbane, B.; Cohen, Y.; Lafarge, A.; Contou, D.; Thille, A.W.; Galerneau, L.M.; Reizine, F.; Cour, M.; et al. Respiratory multiplex PCR and procalcitonin to reduce antibiotic exposure in severe SARS-CoV-2 pneumonia: A multicentre randomized controlled trial. *Clin. Microbiol. Infect.* **2023**, *29*, 734–743. https://doi.org/10.1016/j.cmi.2023.01.009.
26. Sathitakorn, O.; Chansirikarnjana, S.; Jantarathaneewat, K.; Weber, D.J.; Warren, D.K.; Apisarnthanarak, P.; Tantiyavarong, P.; Apisarnthanarak, A. The role of procalcitonin and Clinical Pulmonary for Infection Score (CPIS) score to reduce inappropriate antibiotics use among moderate to severe coronavirus disease 2019 (COVID-19) pneumonia: A quasi-experimental multicenter study. *Infect. Control. Hosp. Epidemiol.* **2023**, *44*, 1199–1203. https://doi.org/10.1017/ice.2022.201.
27. Sibani, M.; Canziani, L.M.; Tonolli, C.; Armellini, M.; Carrara, E.; Mazzaferri, F.; Conti, M.; SAVE Working Group; Mazzariol, A.; Micheletto, C.; et al. Antimicrobial Stewardship in COVID-19 Patients: Those Who Sow Will Reap Even through Hard Times. *Antibiotics* **2023**, *12*, 1009. https://doi.org/10.3390/antibiotics12061009.
28. Spernovasilis, N.; Kritsotakis, E.I.; Mathioudaki, A.; Vouidaski, A.; Spanias, C.; Petrodaskalaki, M.; Ioannou, P.; Chamilos, G.; Kofteridis, D.P. A carbapenem-focused antimicrobial stewardship programme implemented during the COVID-19 pandemic in a setting of high endemicity for multidrug-resistant Gram-negative bacteria. *J. Antimicrob. Chemother.* **2023**, *78*, 1000–1008.
29. Giannella, M.; Rinaldi, M.; Tesini, G.; Gallo, M.; Cipriani, V.; Vatamanu, O.; Campoli, C.; Toschi, A.; Ferraro, G.; Horna, C.S.; et al. Predictive model for bacterial co-infection in patients hospitalized for COVID-19: A multicenter observational cohort study. *Infection* **2022**, *50*, 1243–1253.
